# Hamstring tendon autografts do not show complete graft maturity 6 months postoperatively after anterior cruciate ligament reconstruction

**DOI:** 10.1007/s00167-018-5033-0

**Published:** 2018-07-14

**Authors:** Marcus Hofbauer, Francesco Soldati, Pavol Szomolanyi, Siegfried Trattnig, Francesca Bartolucci, Freddie Fu, Matteo Denti

**Affiliations:** 1 Clinica Luganese, Lugano, Switzerland; 2 IRCCS Galeazzi Orthopaedic Institute, Milan, Italy; 3 Department of Orthopaedic Surgery, University of Pittsburgh, Pittsburgh, PA USA; 4 Department of Orthopaedic and Trauma Surgery, Medical University of Vienna, Waehringerguertel 18-20, 1090 Vienna, Austria; 5 Centre of Excellence “High-field Magnetic Resonance (MR)”, Medical University Vienna, Vienna, Austria

**Keywords:** ACL reconstruction, Hamstring tendon, MRI, Graft healing

## Abstract

**Purpose:**

In this prospective, double-center cohort study, we aim to assess how the anterior cruciate ligament (ACL) signal intensity on magnetic resonance imaging (MRI) potentially varies between a group of patients with anatomic ACL reconstruction using autogenous hamstring grafts 6 months postoperatively and a healthy ACL control group, and how MRI-based graft signal intensity is related to knee laxity.

**Methods:**

Sixty-two consecutive patients who underwent ACL reconstruction using quadrupled hamstring tendon autograft were prospectively invited to participate in this study, and they were evaluated with MRI after 6 months of follow-up. 50 patients with an MRI of their healthy ACL (Clinica Luganese, Lugano, Switzerland) and 12 patients of their contralateral healthy knee (Department of Orthopaedic and Trauma Surgery, Medical University of Vienna, Austria) served as the control group. To evaluate graft maturity, the signal-to-noise quotient (SNQ) was measured in three regions of interest (ROIs) of the proximal, mid-substance and distal ACL graft and the healthy ACL. KT-1000 findings were obtained 6 months postoperatively in the ACL reconstruction group. Statistical analysis was independently performed to outline the differences in the two groups regarding ACL intensity and the correlation between SNQ and KT-1000 values.

**Results:**

There was a significant difference in the mean SNQ between the reconstructed ACL grafts and the healthy ACLs in the proximal and mid-substance regions (*p* = 0.001 and *p* = 0.004). The distal region of the reconstructed ACL showed a mean SNQ similar to the native ACL (n.s). Patients with a KT-1000 between 0 and 1 mm showed a mean SNQ of 0.1; however, a poor correlation was found between the mean SNQ and KT-1000 findings, probably due to the small sample size of patients with higher laxity.

**Conclusion:**

After 6 months of follow-up, hamstring tendon autografts for anatomic ACL reconstruction do not show equal MRI signal intensity compared to a healthy ACL and should therefore be considered immature or at least not completely healed even if clinical laxity measurement provides good results. However, in the case of a competitive athlete, who is clinically stable and wants to return to sports at 6 months, performing an MRI to confirm the stage of graft healing might be an option.

**Level of evidence:**

Prospective, comparative study II.

## Introduction

Techniques in anterior cruciate ligament (ACL) reconstruction have evolved tremendously over the last decade; however, high re-rupture rates remain a concern, with secondary graft failure occurring in 6–25% in young, active patients [11, 17]. To prevent graft re-injury, safe return to sports (RTS) foremost at the “right” time point, when the reconstructed graft has completely healed, is one important factor for young and active people following ACL reconstruction.

In recent years, literature has suggested the use of biological autografts for ACL reconstruction especially in young patients, due to their potential for remodeling, tendon to bone healing and advances compared to allografts [16]. Among the available graft options, hamstring tendon autografts with doubled semitendinosus tendon (ST) and gracilis tendon (GT) have become the most commonly used type for ACL reconstruction nowadays [15], given the fact of a greater mechanical strength compared to a bone–patellar tendon–bone complex and reduced donor site morbidity [6, 13]. Nevertheless, biological processes that occur during graft maturation influence mechanical properties of the knee joint after ACL reconstruction and, therefore, determine the rehabilitation and timing until normal function of the knee joint can be expected [4, 8].

Magnetic resonance imaging (MRI) following ACL reconstruction plays an important role to evaluate the changing appearance of the reconstructed tendon tissue during the healing period; however, no consensus has been found so far on graft signal visibility and prediction of graft maturation using plain or enhanced MRI [5, 12]. Previous animal studies demonstrated a significant correlation between the graft signal intensity measured with MRI and structural properties, but there is still little evidence regarding the relationship between graft signal intensity and the time from surgery [1, 19]. This is crucial, however, to understand which rehabilitation activities might lead to excessive ACL tensioning and therefore should be avoided during the first 6 months, especially in accelerated protocols.

Therefore, the purpose of this study was to compare the potential MRI-based graft signal-to-noise quotient (SNQ) differences between hamstring tendon autografts in a group of patients 6 months after anatomic ACL reconstruction and a matched-compared control group of patients with a healthy ACL. The first hypothesis was that there would be a significant difference of the SNQ between the reconstructed ACL grafts and healthy ACLs 6 months postoperatively. The second hypothesis was that there would be a poor correlation between the SNQ and the laxity of the ACL reconstructed knees.

## Materials and methods

This was a prospective, double-center cohort study performed at the (Clinica Luganese, Lugano, Switzerland) under the scientific supervision of the (IRCCS Galeazzi Orthopedic Institute Milan, Italy) and the (Department of Orthopaedic Surgery, Medical University Vienna, Austria).

Between 2015 and 2016, 62 consecutive patients who underwent anatomic ACL reconstruction were enrolled in this study, with 50 patients at the (Clinica Luganese, Lugano, Switzerland) and 12 patients at the (Department of Orthopaedic and Trauma Surgery, Medical University of Vienna, Austria). At the (Clinica Luganese, Lugano, Switzerland), 50 patients with a healthy ACL who did not undergo surgery served as the control group. The two groups were comparable by age and sex. For the 12 patients that underwent ACL reconstruction in (Department of Orthopaedic and Trauma Surgery, Medical University of Vienna, Austria), their contralateral healthy knee served as the control group. The following inclusion criteria were used: (1) unilateral, isolated ACL injury, (2) ACL reconstruction using a four-stranded hamstring tendon autograft, (3) closed growth plates and less than 50 years of age at the time of surgery, (4) healthy contralateral knee, and (5) no prior injuries to the knee undergoing surgical repair. The following exclusion criteria were used: (1) previous surgery to the operated knee, (2) posterior cruciate ligament (PCL), lateral collateral ligament, or medial collateral ligament injuries superior to grade 2, and (3) cartilage damage or signs of osteoarthritis stage > 2 according to the Outerbridge classification.

### Surgical procedure

The same surgical technique, fixation method, and postoperative protocol were used in both academic centers. In the ACL reconstruction group, each patient was treated by a senior surgeon (M.H and M.D) specializing in knee arthroscopy and with the same arthroscopic technique according to the concept of individualized ACL surgery [7].

Following diagnostic arthroscopy, associated meniscal and cartilage injuries were addressed prior to ACL reconstruction. The semitendinosus and gracilis tendons were harvested and prepared as a four-stranded double-looped autograft. Following identification of the femoral ACL footprint, according to the bony landmarks and remnant fiber position, the femoral tunnel was placed in the center of the anatomic ACL footprint. The tibial tunnel was created at the center of the tibial ACL footprint using a commercially available tibial guide (Smith & Nephew Endoscopy, Andover, MA, USA) set at 55° and then drilled with a cannulated reamer. The graft was then passed through the tibial tunnel, across the joint, and into the femoral tunnel. The femoral part of the graft was fixed using a cortical device (Endobutton CL, Smith & Nephew, Andover, MA, USA). After the graft was tensioned several times, the tibial part of the graft was then fixed using an absorbable interference screw. The graft was fixed to the tibia at 15° of knee flexion. All patients participated in the same standardized postoperative rehabilitation protocol.

### MRI scan and image analysis

At the (Clinica Luganese, Lugano, Switzerland), all images were performed with a 1.5-T MRI scanner (Somatom, Siemens AG, Germany). Standard oblique coronal proton density-weighted images (PDWI) with 3 mm slice thickness were used for analysis of graft maturity. The coronal oblique imaging plane was defined parallel to the Blumensaat’s line. The repetition time (TR) was 2000–2223.2 ms and echo time (TE) was 18 and 95 ms for PDWI and T2WI, respectively.

**Fig. 1:**
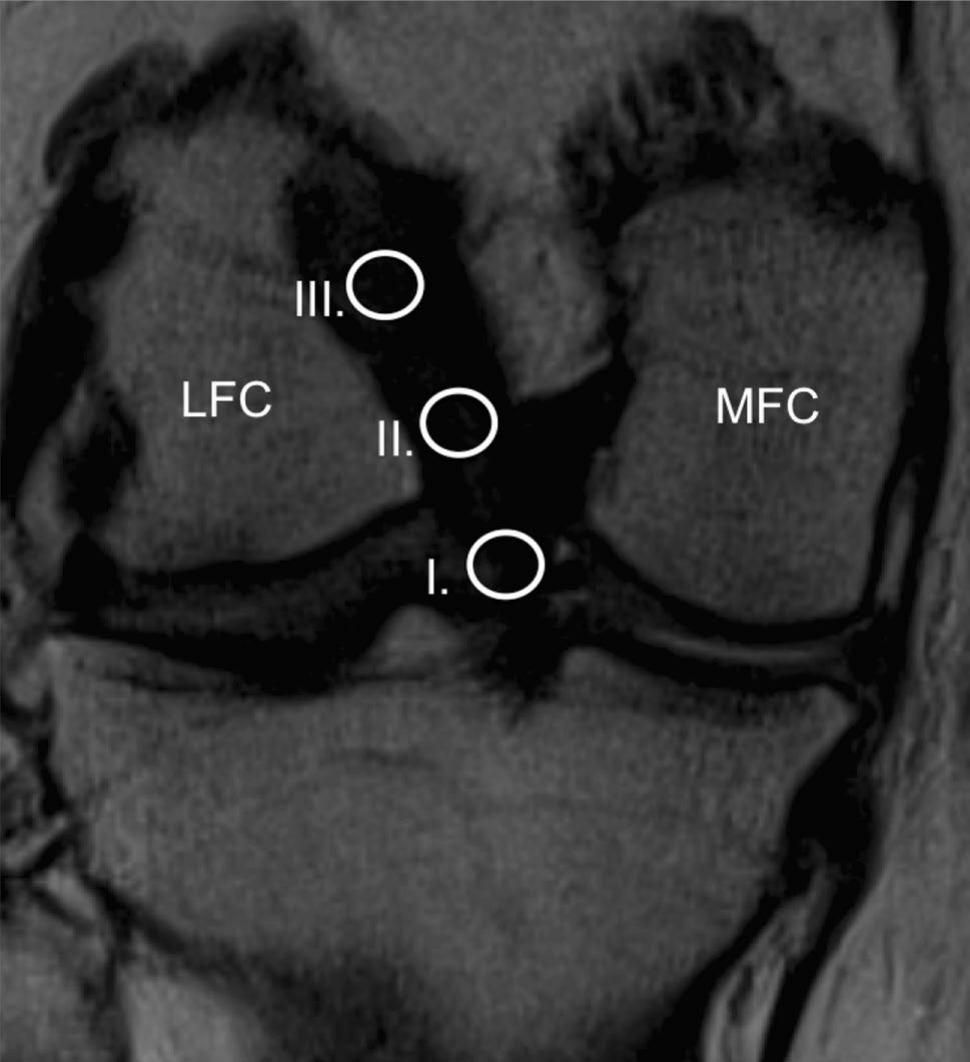
Coronal oblique magnetic resonance imaging scan of a right knee showing the position of the three regions of interest (area of the circle = 0.2 cm^2^), including a (I) distal, (II) middle and (III) proximal site of the ACL. *LFC* lateral femoral condyle, *MFC* medial femoral condyle

Images were obtained from the PACS imaging system and saved as de-identified JPEG images. Using the oblique coronal images, the best single slice that demonstrated the full length of the ACL, including intra-tunnel and intra-articular parts, was selected for analysis **(**Fig. 1**)**. The grafts were evaluated focusing on their gross morphology and signal intensity. The same sequences were obtained to evaluate native ACL in the group of healthy patients.

To assess inter-observer reliability, the images were independently measured in a blinded fashion by two orthopedic radiologists (F.S and F.B). To assess intra-observer reliability, one observer (F.B) made all measurements twice 8 weeks apart.

**Fig. 2:**
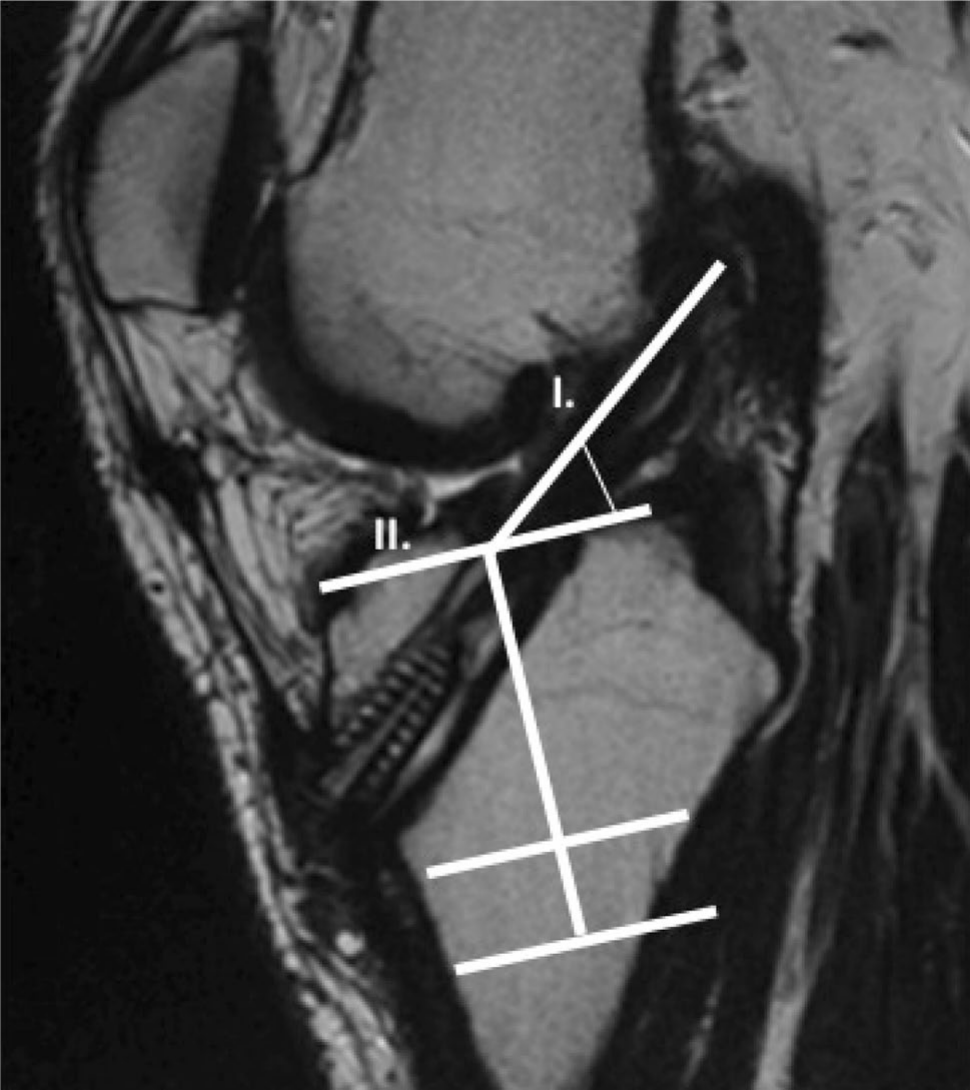
The inclination angle of the ACL, measured on the sagittal view, was defined by the intersection of two lines: one tangential to the anterior aspect of the ACL graft (I) and the other tangential to the anterior aspect of the intercondylar eminence and perpendicular to the long axis of the tibia (II)

To compare the anatomic position of the reconstructed ACL, the graft inclination angle was measured on sagittal MRI views. The sagittal obliquity of the graft was defined by the intersection of two lines: one tangential to the anterior aspect of the graft and the other tangential to the anterior aspect of the intercondylar eminence and perpendicular to the long axis of the tibia, as seen on the MRI image **(**Fig. 2**)**.

At the (Department of Orthopaedic and Trauma Surgery, Medical University of Vienna, Austria), all subjects were scanned on an investigational 7-T whole-body MR scanner (Siemens Healthcare, Erlangen, Germany) running software version VB17A, equipped with a 28-channel transmit-receive knee coil (Quality Electrodynamics, Mayfield Village, OH).

The following sequences were examined in each individual time point for quantitative evaluation of ACL:

1. Sagittal oriented proton density-weighted turbo spin echo with fat suppression.
2. Coronal oriented proton density-weighted turbo spin echo with fat suppression.
3. Parasagittal oriented T2-weighted turbo spin echo.

Sagittal and coronal oriented images provided detailed morphology of the entire knee joint, while parasagittal images were used for SNQ quantification. Parasagittal oriented protocol was set up as follows: the field of- view was 160 × 160 mm, matrix size was 1024 × 1024 pixels, corresponding to the in-plane resolution of 0.156 × 0.156 mm, and slice thickness was 2.5 mm with slice separation of 0.75 mm. The total measurement time was 2 min 15 s, while acquiring 15 slices. The repetition time was set to 5000 ms, echo time was set to 102 ms, and echo train length was 11.

To compare the maturity difference between hamstring tendon autograft and the native ACL, their signal intensity was measured independently for the intra-articular part in the coronal oblique image (Clinica Luganese, Lugano, Switzerland) and parasagittal image (Department of Orthopaedic and Trauma Surgery, Medical University of Vienna, Austria) by using the freehand region-of-interest (ROI) function using JiveX Dicom Viewer (VISUS Health IT GmbH, 44801 Bochum, Germany).

**Fig. 3:**
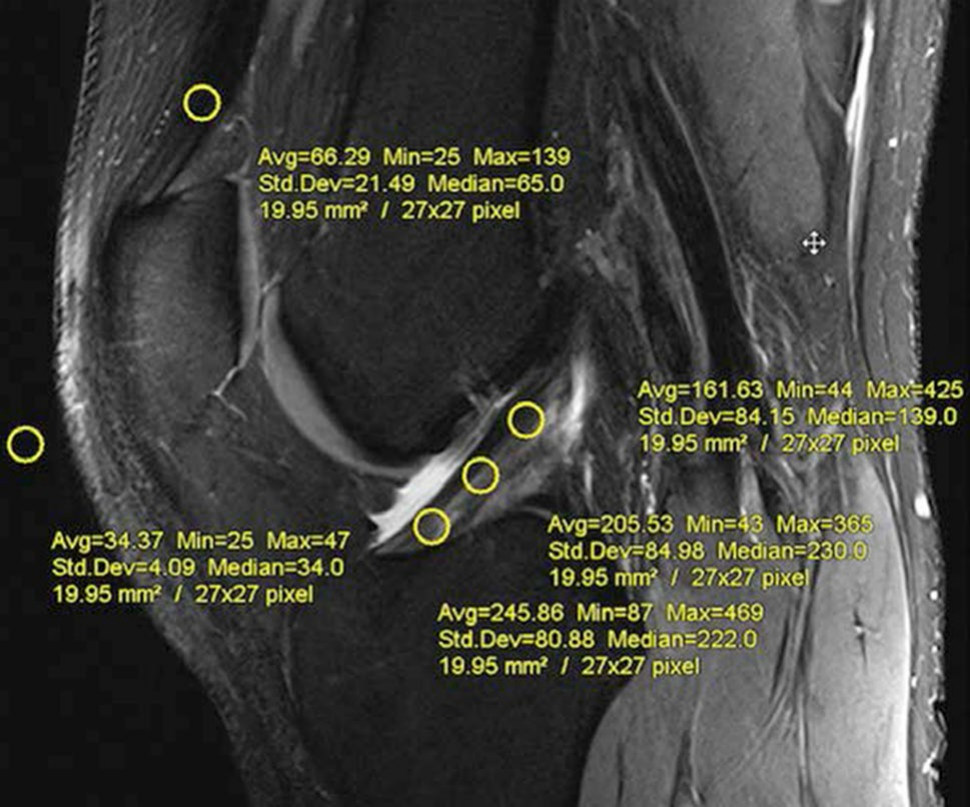
Placement of regions of interest (ROIs) used to calculate the signal-to-noise quotient, measured at the sagittal view. Three ROIs were placed on the graft (tibial, mid-substance, and femoral), one ROI 2 cm proximal of the patella, and one ROI on an empty area 2 cm anterior to the patellar tendon

Circular ROIs 5 mm in diameter were evaluated for measuring the signal of the PCL and background. The background ROI was placed approximately 1 cm medial and 2 cm distal to the medial joint line. Intra-articular graft and native ACL signal intensity were additionally measured independently for three regions in the: (1) proximal region, (2) mid-substance region, and (3) distal region (Fig. 3). The diameter of ROI equals the width of the graft, and the circle of the proximal or distal ROI divided the boundary between the tunnel and notch. The midpoint of the central ROI was located at the midpoint between those of the proximal and distal ROIs. The mean signal intensity and standard deviations were recorded based on image pixels as absolute signal intensity with a measurement accuracy of one decimal. The SNQ was calculated for each graft with the following formula [8]:

$${\text{SN}}{{\text{Q}}_{{\text{ACL}} - {\text{tissue of interest}}}}={\text{ }}\left( {{\text{SIGNA}}{{\text{L}}_{{\text{ACL}} - {\text{tissue of interest}}}} - {\text{ SIGNA}}{{\text{L}}_{{\text{PCL}}}}} \right)/{\text{SIGNA}}{{\text{L}}_{{\text{background}}\_{\text{noise}}}}.$$

Lesser SNQ ratios are supposed to indicate lesser water content and theoretically better maturity and healing of the graft.

### Clinical evaluation

Six months postoperatively, the KT-1000 knee arthrometer (MED-metric Corp, San Diego, California, USA) was used to measure the side-to-side difference in the anterior tibial translation in the ACL reconstructed group. An experienced orthopedic surgeon (F.S, M.H) performed all physical examinations.

The institutional review board (Medical University of Vienna, IRB: 1562–2014) approved the study and written informed consent was obtained from all subjects.

### Statistical analysis

The Student’s *t* test and Mann–Whitney *U* test were used to assess differences between the two groups (operated and healthy patients) regarding SNQ values and ACL angle. The variance analysis and Friedmann test were used to compare the three portions of the intra-articular part of the ACL in the same subjects. Pearson and Spearman correlation coefficients were used to determine the relationship between the mean SNQ of the ACL graft and the knee laxity at the KT-1000 arthrometer. Intra-observer and inter-observer agreement for signal intensity were evaluated using intra-class correlation coefficient with 95% confidence interval (95% CI). Statistical significance was defined as *p* < 0.05. All data were analyzed using SPSS version 16 (SPSS Inc, Chicago, Illinois, USA).

## Results

At the (Clinica Luganese, Lugano, Switzerland), there was a significant difference in the mean SNQ between the reconstructed ACL grafts and the healthy ACL in the proximal and middle intra-articular portions (*p* = 0.001 and *p* = 0.004) at 6 months of follow-up. The distal region of the reconstructed ACL showed a mean SNQ similar to the native ACL (n.s). However, the mean SNQ of HS grafts (n.s) was found to be homogeneous among the three portions without any significant difference.

There was no significant difference of the ACL graft angle measurement between the reconstructed ACL and healthy ACL, with 49.2° ± 3.5° and 50.1° ± 3.1°, respectively. The intra-observer and inter-observer agreement for the signal intensity of the ROI was excellent with 0.995: CI 0.991–0.998 and 0.994: CI 0.990–0.998, respectively.

At the (Department of Orthopaedic and Trauma Surgery, Medical University of Vienna, Austria), there was a significant difference in the mean SNQ between the reconstructed ACL grafts and the contralateral, healthy ACLs in all portions (*p* ≤ 0.05) at 6 months of follow-up.

**Fig. 4:**
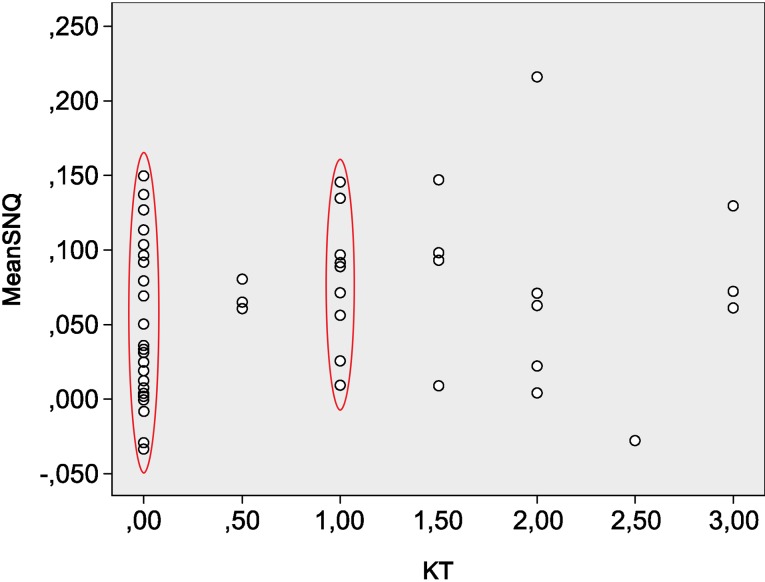
In the ACL-reconstructed group, the dispersion graph shows that the mean ATT measurement with the KT-1000 arthrometer is 0.7 mm (0–3 mm). Patients with an ATT between 0 and 1 mm had a mean SNQ of 0.1, but a poor correlation was found between mean SNQ and KT-1000 findings, both with Pearson and Spearman coefficients. In fact, the majority of cases (73% of the operated patients) were concentrated in the range between 0 and 1 mm, while only 8% of patients had a borderline value (3 mm) of laxity

In the ACL-reconstructed group, the mean ATT measurement with the KT-1000 arthrometer was 0.7 mm (0 to 3 mm). Patients with an ATT between 0 and 1 mm showed a mean SNQ of 0.1, but a poor correlation was found between mean SNQ and KT-1000 findings both with Pearson and Spearman coefficients. The dispersion graph also shows that the majority of cases (73% of the operated patients) were concentrated in the range between 0 and 1 mm, while only 8% of patients had a borderline value (3 mm) of laxity **(**Fig. 4**)**.

## Discussion

The main finding of this study is the significant difference of the SNQ value in the proximal and mid-substance region between the reconstructed ACL grafts and the control group with healthy ACLs at 6 months of follow-up.

These observations of a higher SNQ value in the proximal part of the ACL are consistent with previously reported findings of MRI-based graft healing [2, 10, 17, 18]. Ma et al. [10] compared the MRI findings between the quadriceps tendon with bone block and hamstring tendon autografts 6 months after primary ACL reconstruction. For hamstring tendon autograft, the authors found that the distal region showed a significantly lower mean SNQ value compared to the mid-substance and proximal region, thus indicating a better maturity in the distal region. In contrast, the mean SNQ of the proximal region in the quadriceps tendon with bone block graft group was lesser compared to the hamstring group. This difference could be explained by the fact that in the quadriceps group a bone block is inserted in the femoral tunnel where bone-to-bone healing takes place and has been shown to provide better and faster healing than tendon-to-bone healing [14].

Tashiro et al. [17] evaluated the SNQ in three different regions (proximal/mid-substance/distal) in 24 patients that underwent primary ACL reconstruction using an autologous quadriceps tendon with bone plug. At 6 months follow-up, the SNQ of the ACL graft in the proximal region was significantly higher than in the mid-substance and distal regions. Two years postoperatively, signals in the proximal and mid-substance regions decreased significantly compared with 6 months and no statistical difference was found among the three regions at 24 months. The authors suggest that the steeper graft bending angle (GBA) on the femoral side, compared with the traditional transtibial technique, may contribute to increased ACL graft signals in the proximal regions.

Chen et al. [2] showed similar findings with the GBA having a significant positive association with graft SNQ in the femoral tunnel or proximal site following ACL reconstruction with either allograft or autograft tendons.

The GBA is defined as the angle between the femoral bone tunnel and the line connecting the femoral and tibial tunnel apertures. Compared to the tibial tunnel aperture, where the ACL graft is almost a straight line, it bends steeply on the femoral side. It has been assumed that the abrasive force and repetitive bending at the sharp femoral aperture could cause excessive stress on the bone–graft interface and thus prolong graft healing [17].

On the other hand, the distal regions of the ACL graft had a lower mean SNQ value, suggesting a more advanced healing at the tibial side. One explanation for this finding could be the presence of microvessels from the infrapatellar fat pad improving the perfusion at the graft site [9]. These present results indicate that 6 months postoperatively, hamstring tendon grafts do not show a sufficient level of maturity. Therefore, it may be advisable to slow the progression of activities in individuals with HS autografts at this time, especially for athletes ready to be cleared for returning to competition—activities for which the graft may not be mature enough.

The second hypothesis was that there would be a correlation between a higher SNQ value of the ACL graft and clinical knee laxity; however, a poor correlation was found between the mean SNQ and KT-1000 arthrometer findings, probably because of the lack of cases with higher laxity. Future studies should also investigate this relationship between SNQ and graft strength.

There are limitations to the present study. First, the SNQ measurement method of MRI images is highly variable depending on multi-element surface coils, parallel imaging, and different reconstruction filters. For example, the noise distribution in parallel imaging is described by the spatially varying geometry factor (*g*-factor) and depends on parameters such as the coil geometry, phase-encoding direction, and acceleration factor. In this case, the determination of the noise intensity using a background ROI may lead to inaccurate results and thus to over- or underestimation of SNR [3]. Another limitation is the relative short follow-up time of 6 months. However, current rehabilitation guidelines still enable patients to return to demanding sports-specific activities within 6 months postoperatively. Considering the current findings of an increased SNQ, specifically at this period of time, physicians should be aware that the graft maturity might not be complete and clearing patients back to contact sports would increase the risk for graft re-rupture. However, in the case of a competitive athlete, who is clinically stable and wants to return to sports at 6 months, performing an MRI to confirm the stage of graft healing might be an option.

## Conclusions

At 6 months of follow-up, hamstring tendon autografts for anatomic ACL reconstruction do not show the same MRI signal intensity compared to a healthy ACL and should therefore be considered immature or at least not fully healed even if clinical laxity measurement provides good results. These findings suggest that the criteria for returning to demanding sports after ACL reconstruction should not be considered based on the functional stability and muscle strength alone.
